# Receptor-mediated signaling in *Aspergillus fumigatus*

**DOI:** 10.3389/fmicb.2013.00026

**Published:** 2013-02-20

**Authors:** C. M. Grice, M. Bertuzzi, E. M. Bignell

**Affiliations:** South Kensington Campus, Imperial College LondonLondon, UK

**Keywords:** *Aspergillus fumigatus*, stress, virulence, signaling

## Abstract

*Aspergillus fumigatus* is the most pathogenic species among the Aspergilli, and the major fungal agent of human pulmonary infection. To prosper in diverse ecological niches, Aspergilli have evolved numerous mechanisms for adaptive gene regulation, some of which are also crucial for mammalian infection. Among the molecules which govern such responses, integral membrane receptors are thought to be the most amenable to therapeutic modulation. This is due to the localization of these molecular sensors at the periphery of the fungal cell, and to the prevalence of small molecules and licensed drugs which target receptor-mediated signaling in higher eukaryotic cells. In this review we highlight the progress made in characterizing receptor-mediated environmental adaptation in *A. fumigatus* and its relevance for pathogenicity in mammals. By presenting a first genomic survey of integral membrane proteins in this organism, we highlight an abundance of putative seven transmembrane domain (7TMD) receptors, the majority of which remain uncharacterized. Given the dependency of *A. fumigatus* upon stress adaptation for colonization and infection of mammalian hosts, and the merits of targeting receptor-mediated signaling as an antifungal strategy, a closer scrutiny of sensory perception and signal transduction in this organism is warranted.

## Introduction

The genus *Aspergillus* is comprised of environmental filamentous mold fungi which utilize decaying organic matter for metabolic energy and nutrition. *Aspergillus fumigatus* is the most pathogenic, and is commonly isolated as an agent of human pulmonary infections (Dagenais and Keller, 2009). In healthy individuals, mucociliary clearance and pulmonary immune defences clear the hundreds of conidia inhaled daily (Balloy and Chignard, 2009). However, medical advances in transplantation and anticancer therapies have expanded the immunosuppressed patient population, and the number of individuals infected by opportunistic organisms, such as *A. fumigatus*, has drastically increased (McNeil et al., 2001; Chamilos et al., 2006). For opportunistic fungal pathogens, the phenomenon of “ready-made” virulence has been postulated, whereby traits which evolved for survival in ecological niches also govern survival in susceptible immuno-compromised hosts (Casadevall et al., 2003; Rhodes, 2006).

**Figure 1:**
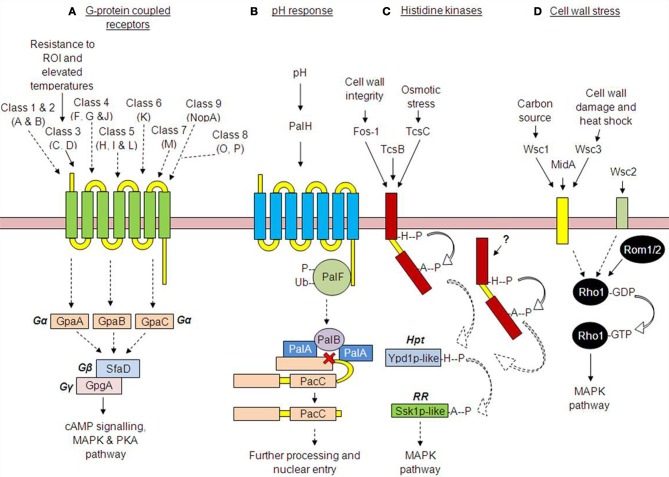
**Receptor-mediated signaling in *Aspergillus fumigatus*. (A)** *G-protein coupled receptor pathways*—So far 15 GPCRs have been identified in *A. fumigatus*, though only two have been characterized (GprC and D). **(B)** The *pH-response pathway*—A shift from acidic to alkaline environmental pH is thought to be detected by the receptor PalH, mediating the phosphorylation, and subsequent ubiquitination of the C-terminally bound arrestin, PalF. This stimulates the proteolytic cleavage of the transcription factor PacC. PacC then undergoes further pH independent cleavage, before translocating to the nucleus. **(C)** *The histidine kinase receptor pathway—*The phosphorelay histidine kinases mediate the transduction of specific external or cytosolic stimuli such as cell wall integrity (Fos-1) and osmotic stress (TcsC), stimulating autophosphorylation upon a conserved histidine residue. The activating stimulus for TcsB remains elusive. **(D)** *The cell wall stress pathway—*Cell wall receptors detect environmental stress such as cell wall damage/heat shock (MidA) and alternative carbon sources (Wsc1); however, the specific stimuli for receptors Wsc2 and 3 remain elusive. (Dotted lines indicate predictions based on studies in other fungi).

Beyond residual host immune responses, there are additional obstacles to successful colonization of the mammalian lung, including tolerance of host-facilitated stresses, such as iron starvation (Schrettl et al., 2004, 2007) and alkaline pH (Peñalva and Arst, 2004; Bignell et al., 2005; Peñalva et al., 2008). The requirement for infecting fungi to detect and respond to such extracellular cues is often essential for infectious growth, and in *A. fumigatus* the fungal receptors through which the extracellular environment is sensed remain largely unknown. This review discusses current knowledge on receptor-mediated signaling in *A. fumigatus* (Figure 1) and catalogues all of the putative seven transmembrane domain (7TMD) sensors encoded by the *A. fumigatus* genome (Table A1). Our analysis exposes the vast numbers of uncharacterized *A. fumigatus* receptor-like proteins.

## G protein coupled receptors (GPCRs) in *A. fumigatus*

*In silico* analyses of fungal genome sequences have identified genes encoding putative GPCR proteins. In the phytopathogenic fungus *Magnaporthe grisea*, a screen of the predicted proteome using all GPCR sequences at the time available in the GPCR Database (GPCRDB) (Horn et al., 2003) yielded 14 GPCR-like sequences (Kulkarni et al., 2005). A similar exercise applied to *A. fumigatus* identified 15 putative GPCRs (Lafon et al., 2006).

In Aspergilli, putative GPCRs are classified by homology, and according to a convention established by Lafon et al. (2006) in *A. nidulans*, into nine groupings. In *A. fumigatus*, Classes 1 and 2 are comprised, respectively, of two putative pheromone receptors GprA (AFUA_3G14330) and GprB (AFUA_5G07880); Class 3 is comprised of two putative carbon sensors GprC (AFUA_7G04800), GprD (AFUA_2G12640); Class 4 is comprised of three putative nitrogen sensors GprF (AFUA_5G04100), GprG (AFUA_1G11900), and GprJ (AFUA_1G06840); Class 5 of three putative cAMP receptors GprH (AFUA_5G04140), GprI (AFUA_3G00780), and GprL (AFUA_ 3G01750), the latter being unique to *A. fumigatus*; Class 6 is comprised of a single putative GPCR, GprK (AFUA_4G01350) having a regulator of G-protein signaling (RGS) domain, unique to filamentous fungi; Class 7 includes two putative GPCRs with homology to rat growth hormone-releasing factor receptors (Miller et al., 1999) only one of which is found in *A. fumigatus*, GprM (AFUA_7G05300); Class 8 is comprised of three putative GPCRs with identity to yeast Izh zinc regulators (Karpichev et al., 2002; Lyons et al., 2004), two of which are found in *A. fumigatus* GprO (AFUA_ 3G10570) and GprP (AFUA_6G07160), and Class 9 is comprised of a single putative GPCR, NopA (AFUA_7g01430) having identity to bacterial opsins. The roles of some of these receptors have been identified in other species though in *A. fumigatus* little is known (Figure 1).

Among the 15 predicted GPCR-like proteins in *A. fumigatus*, only two, GprC (AFUA_7G04800) and GprD (AFUA_2G12640), have been characterized (Gehrke et al., 2010). GprC and GprD have been noted as having homology to Gpr1p of *Saccharomyces cerevisiae* which activates the cAMP pathway in response to glucose, as demonstrated by cAMP enzyme immunoassay (Yun et al., 1998; Kraakman et al., 1999). Furthermore, the *A. nidulans* GprD homologue mediates increase of intracellular cAMP in response to oxygenated polyunsaturated fatty acids (oxylipins), which act as autocrine and paracrine mediators in eukaryotic organisms (Affeldt et al., 2012). Deletion of *A. fumigatus* GprC and GprD resulted in significant growth impairment under all tested growth conditions and analysis of virulence revealed significant attenuation of virulence for *ΔgprD* and delayed mortality for *ΔgprC* in a murine model of aspergillosis (Gehrke et al., 2010). The remainder of the putative *A. fumigatus* GPCRs remain to be investigated and nothing is known about their molecular linkages to multi-subunit G-proteins. Unlike most *Aspergillus spp*. where four predicted Gα subunits occur, only three (GpaA, AFUA_1G13140, GpaB, AFUA_1G12930, and GpaC, AFUA_3G12400) have been identified for *A. fumigatus* (Liebmann et al., 2003), which presumably act via interaction with the Gβ and Gγ subunits (SfaD, AFUA_5G12210 and GpgA, AFUA_1G05210). In the current absence of other identified G protein subunits, or similar proteins, it is thought that the aforementioned five proteins service the entire *A. fumigatus* GPCR repertoire (Figure 1). Undoubtedly the relevance of *A. fumigatus* Gβ and Gγ subunits for viability and vegetative growth is significant as *ΔsfaD* and *ΔgpgA* gene deletion mutants are extremely impaired for germination and vegetative growth (Shin et al., 2009).

## Genome-wide *in silico* predictions of *A. fumigatus* integral membrane proteins

Kulkarni et al. (2005) noted, based upon membrane topology, that the number of putative GPCR-like proteins encoded by the *M. grisea* genome rose to 76 when the criteria were relaxed to include homologs of the Pth11 receptor (DeZwaan et al., 1999) which is required for *M. grisea* pathogenicity in rice. Applying a more universal approach to *A. fumigatus*, we used the published genome sequence (Nierman et al., 2005) to catalogue all *A. fumigatus* proteins having predicted TMDs (Figure 1). To implement this, we used the TMPRED (Hofmann and Stoffel, 1993) predictive tool to perform an analysis of all 9497 *A. fumigatus* proteins encoded by the reference genome Af293 (Nierman et al., 2005) http://www.cadre-genomes.org.uk/Aspergillus_fumigatus/Info/Index. In total we identified 6496 proteins having putative TMDs. Among them, 161 proteins were found to encode seven predicted TMDs (Tables 1 and A1). The majority of the predicted 7TMD proteins are of hypothetical function (Table A1).

**Table 1:** **Numbers of predicted *A. fumigatus* TMD proteins, by chromosome**.

**TMDs**	**Chromosome number**							
	**1**	**2**	**3**	**4**	**5**	**6**	**7**	**8**
0	557	519	424	398	428	358	179	138
1	311	404	378	251	288	282	179	161
2	267	257	228	199	206	194	106	114
3	127	233	100	100	141	116	51	57
4	81	85	59	60	56	57	25	40
5	132	38	23	3	22	31	18	17
6	22	26	17	21	16	21	14	8
7	18	22	23	19	28	27	7	16
8	9	10	16	9	10	5	5	4
9	19	13	12	13	15	15	4	9
10	15	23	20	13	16	7	7	12
11	22	22	23	29	21	16	9	18
12	23	25	24	17	18	19	12	9
13	7	6	9	8	7	8	2	1
14	3	2	6	3	5	6	2	3
15	3	1	4	1	2	2	1	1
16	0	1	2	0	2	4	0	1
17	0	0	0	0	1	1	0	0
18	0	0	0	2	1	0	0	0
19	0	0	0	0	0	0	0	0
20	0	0	0	0	0	0	0	0
21	0	0	0	0	0	0	0	0
22	1	0	0	0	0	0	0	0

## PalH: a putative 7TMD pH sensor

During colonization of the mammalian lung *A. fumigatus* is exposed to a range of microenvironments, of likely differing pHs, not only within the pulmonary niche but also following phagocytosis by macrophages or ingestion by neutrophils and exposure to their vacuole contents (Levitz et al., 1999; Newman, 1999; Reeves et al., 2002; Ibrahim-Granet et al., 2003). Versatility of metabolism and physiology is required to survive such extremes, including appropriate pH-responsive gene expression for nutrient acquisition and survival (Bignell et al., 2005). In the model ascomycete and occasional pathogen *A. nidulans*, the PacC transcription factor governs gene expression in response to extracellular pH (Tilburn et al., 1995; Diez et al., 2002) and is vital for mammalian pathogenicity (Peñalva and Arst, 2004; Bignell et al., 2005; Peñalva et al., 2008). Under alkaline conditions, a signaling cascade involving seven proteins is involved in activation of PacC. A putative pH sensor, PalH, has 7TMDs and a cytoplasmic C-terminus (Negrete-Urtasun et al., 1997, 1999), which interacts with a cognate arrestin encoded by *palF* (Herranz et al., 2005; Hervas-Aguilar et al., 2010). Unlike canonical GPCR receptors, PalH is not thought to act via interaction with G-protein subunits (Kroeze et al., 2003). When an alkaline response is triggered, PalF is phosphorylated and subsequently ubiquitinated in a PalH-dependent manner (Herranz et al., 2005), leading to PalB-mediated, signal dependent, proteolytic cleavage of the pH-responsive transcription factor PacC (Penas et al., 2007; Rodriguez-Galan et al., 2009). Subsequent translocation of the truncated PacC protein, from cytoplasm to nucleus, permits alkaline adaptation via differential expression of genes required to enable growth under alkaline extracellular conditions (Tilburn et al., 1995; Mingot et al., 1999, 2001; Espeso and Arst, 2000; Espeso et al., 2000). In *A. fumigatus* the amino acid residues crucial for PalH and PalF interaction are conserved, and in split-ubiquitin analyses the proteins enter into close proximity (Bertuzzi and Bignell, 2011; Bignell, 2012). We have also recently demonstrated the requirement for *A. fumigatus* PalH for murine infection (Bertuzzi et al., in preparation).

## Histidine kinase sensors in *A. fumigatus*

Histidine kinases (HK) are phosphorelay protein sensors which transduce extracellular signals. HKs are common in the fungal kingdom, and apparently absent in humans (West and Stock, 2001). Amongst archaea, bacteria and fungi, two classes of HK (two-component and hybrid) are found. The former class of two-component receptor systems predominate in bacteria and archaea, whereby autophosphorylation of the HK protein precedes transfer of the phosphoryl group to a conserved aspartate residue in a second protein, termed the response regulator (RR)(Li et al., 2012). HK activities have been associated with both the osmo- and peroxide-regulatory pathways in multiple fungi, and have been most extensively characterized in *S. cerevisiae* (Santos and Shiozaki, 2001). However, RR proteins are not abundantly encoded by fungal genomes; Skn7 and Ssk1 are two examples of such proteins, which in *S. cerevisiae* and *C. albicans*, account for the entire RR cohort of these species (Kaserer et al., 2009; Oide et al., 2010). The fungal phosphotransfer relay can involve three proteins, as exemplified by the *S. cerevisiae* HOG1 MAPK phosphorelay, where an HK (Sln1), a histidine phosphointermediate (Ypd1) and an RR (Ssk1) collectively mediate a multistep phosphotransfer (Kaserer et al., 2009).

Fungal HKs most commonly fall into the hybrid class of regulators which utilize a single polypeptide. This protein possesses both a Histidine kinase A (HiskA) and a receiver domain (REC) containing a conserved aspartate residue (Li et al., 2012). Other domains, such as the ATP-binding HATPase_c domain (Dago et al., 2012) are also found; however, as these proteins are largely uncharacterized for *A. fumigatus*, the functional relevance of domain organization is unknown. The composition, and/or relative positioning, of additional domains provides the basis for sub-classification of HKs (Catlett et al., 2003), presented for 12 *A. fumigatus* HKs in Table A2. Amongst these, only three have been studied: the two-component system proteins A, B, and C (TcsA/Fos-1 AFUA_5G10240, TcsB AFUA_2G00660 and TcsC AFUA_2G03560).

Despite the significance of the HK Fos-1 for detection of extracellular stresses, this hybrid HK has been previously predicted as possessing no TMDs, implying a cytosolic presence (Pott et al., 2000). However, our TMPRED analyses predicted TMDs for all of the three HKs, with Fos-1 possessing a single TMD (Table A2). Deletion of the *fos-1* gene leads to a ~66% reduction in conidiation after 48 h in liquid YG medium, as well as heightened resistance to the cell wall-degrading enzyme mix novozym 234, suggesting the role of *fos-1* in cell wall assembly (Pott et al., 2000). *Δfos-1* mutants were found to have normal morphology, germination, osmotic and oxidative stress tolerance, and antifungal susceptibilities. Subsequent transcriptional analyses found a significant increase in *fos-1* expression, relative to *in vitro* growth, during the first 72 h of infection in a murine model of pulmonary aspergillosis (Zhang et al., 2005), and reduced virulence of *A. fumigatus* in a systemic murine model of infection (Clemons et al., 2002).

In a study addressing the role of oxidative stress in *A. fumigatus* pathogenicity, Du et al. (2006) characterized the *A. fumigatus* TcsB protein, a putative homolog of Sln1 in *S. cerevisiae*. In *A. nidulans*, TMPRED analysis predicted 2 TMDs for TcsB at the N-terminus (Furukawa et al., 2002), though in *A. fumigatus*, our prediction extends this to 4TMDs (Table A1). Unlike *S. cerevisiae* where deletion of *sln1* is lethal (Maeda et al., 1994), an *A. fumigatus ΔtcsB* mutant is viable, demonstrates normal morphology, and is as tolerant as the wild type to increased temperatures, various cell wall damaging agents, and poor nitrogen/carbon sources. The only phenotype discernable for the mutant was a minor sensitivity to SDS (Du et al., 2006). This data suggests a non-essential role for TcsB, or redundancy of function with other, as yet uncharacterized protein(s).

It is believed that group III HK mediate resistance to high osmotic pressure via the high osmolarity glycerol pathway (HOG). For this reason, the characterization of the sole *A. fumigatus* group III hybrid HK TcsC, classified as such on the basis of putative HAMP (HK, adenyl cyclase, methyl-accepting chemotaxis protein, phosphatase) domains, was investigated (McCormick et al., 2012). The significance of the HAMP domains, based upon studies of other sensor proteins and signaling is postulated as providing the means to couple input and output since HAMP domains of integral membrane hybrid HKs are found in close proximity to the membrane-spanning segment (Parkinson, 2010). It is speculated that in response to extracellular signals, such as altered osmolarity, a conformational rearrangement is triggered which prompts activation of an output domain (Parkinson, 2010). In *A. fumigatus*, deletion of the *tcsC* gene resulted in an extended white colony rim and a reduced number of extending hyphae. However, unlike the *A. nidulans* homologue *nikA* (Hagiwara et al., 2009), no detrimental effects on sporulation and conidial growth were observed. In the presence of nitrate as a nitrogen source a significant reduction in radial growth was detectable, and furthermore, compared to the control strain, growth of *ΔtcsC* at 2% O_2_ abolished sporulation and prompted a dome-shaped morphology indicative of oxygen starvation. A strong inhibition of growth resulted from exposure to hyperosmotic stress (1.2 M sorbitol, 1 M KCl, or 1 M NaCl) but sensitivity to calcofluor white, amphotericin B, posaconazole and caspofungin, extremes of pH/temperature, or oxidative stress were reportedly normal.

In a comparative analysis of wild type and *ΔtcsC* virulence, no discernable differences in pathogenicity analysis in a murine model of invasive aspergillosis were detected (McCormick et al., 2012).

## Cell wall receptors

The fungal cell wall is essential for viability and an important target of antifungal drugs (Latgé et al., 2005; Latgé, 2007; Walker et al., 2010). In fungi a conserved MAPK signaling module is responsible for maintaining cellular integrity, shape and resilience to environmental stresses (Lee and Levin, 1992; Levin, 2005; Lesage and Bussey, 2006). In *S. cerevisiae*, such MAPK signaling (Chen and Thorner, 2007) is initiated through stress detection at five integral membrane receptors Wsc1-3, Mid2, and Mtl1 (Lodder et al., 1999). This promotes guanine nucleotide exchange factor (GEFs—Rom1 and Rom2)-mediated nucleotide exchange upon the GTPase Rho1, facilitating the regulation of numerous downstream effectors (Zu et al., 2001). In a quest to find equivalent cell wall sensors in *A. fumigatus*, Dichtl et al. (2012) performed BLAST analyses to reveal three previously uncharacterized open reading frames with domain structures similar to those of Wsc1-3 (Af Wsc1, AFUA_4G13670, Af Wsc2, AFUA_3G07050, and Af Wsc3, AFUA_5G09020 respectively). Bioinformatic analyses predicted the presence of characteristic cell wall integrity (CWI) sensor N-terminal WSC domains with downstream, though truncated, ser/thr rich regions, and (with the exception of Wsc2) transmembrane domains. In common with the *S. cerevisiae* sensors a short cytosolic C-terminus was also predicted for two of the sensors (Dichtl et al., 2012).

To discern subcellular localization, ectopically integrating vectors were applied to generate four putative CWI sensor-GFP fusions, Wsc1-3, and MidA. From these, localization of all C-terminally tagged sensors was observed at the fungal surface. Additionally a strong presence was observed in vacuoles, though this was dismissed as a by-product of over expression or misfolding. Phenotypic analyses of single and double mutants identified a significant impairment of radial growth in the case of a *Δwsc1Δwsc3* double mutant. These findings were further exacerbated in a triple mutant *Δwsc1Δwsc3ΔmidA*. Furthermore, in all mutants lacking *wsc1*, provision of glycerol as carbon source lead to a significant reduction in radial growth on minimal media (Dichtl et al., 2012).

Previously, mutants lacking members of the CWI MAPK pathway have demonstrated a clear sensitivity to echinocandins and azole antifungals (Fujioka et al., 2007; Dirr et al., 2010). Extending this analysis to the *A. fumigatus* mutant phenotypes revealed a single relevant phenotype, namely the heightened sensitivity of the *Δwsc1* mutant to the echinocandin, caspofungin (Dichtl et al., 2012).

To study stress-induced activation of intracellular signaling, effects on growth and MpkA phosphorylation were analyzed in the presence of the cell wall perturbing agent, calcoflour white, or following heat shock (48°C). None of the Wsc mutants were found to be sensitive to cell wall perturbation or heat shock, however, *ΔmidA* was highly sensitive to all of these stresses. In agreement with phenotypic data, calcofluor white-induced MpkA phosphorylation was significantly reduced in the *ΔmidA* mutant compared with wild type, while phosphorylation of MpkA was not diminished in mutants lacking *wsc1* or *wsc1* and *wsc3* (Dichtl et al., 2012). In *S. cerevisiae*, the Wsc1 cell wall sensor mediates signaling of alkaline stress via the CWI MAPK module (Serrano et al., 2006); no evidence for such a role in *A. fumigatus* was obtained. Thus, while Mkk2 null mutants are sensitive to alkalinization of the medium (Dirr et al., 2010), the identity of the activating cell wall sensor remains unknown.

Taken together these findings suggest that MidA is the sole cell wall perturbation sensor, while Wsc1 is required for glycerol carbon source assimilation. Furthermore, a compensatory role between Wsc1 and Wsc3 with regards to efficient growth and conidiation has been demonstrated. Despite these observations, a role for Wsc2 has yet to be identified, while the putative CWI pH sensor remains elusive.

## Receptor-mediated signaling during *A. fumigatus* infection: relevance for therapeutic strategy

Drugs which target GPCR function account for >50% of currently licensed drugs (Davies et al., 2008). It therefore follows that fungal GPCRs are likely to be similarly responsive to chemical perturbations. This fact, coupled with the absolute requirement for some GPCRs in fungal growth make a compelling case for these proteins as antifungal drug targets. Although the pharmaceutical market is dominated by GPCR-active molecules, the discovery of most of these agents was made on the basis of functional activity in high throughput screens, only later were the targets and modes of action clarified (Filmore, 2004). In the post-genomic era, with confidence in the pharmaceutical relevance of such proteins, drug discovery can become target-driven. An expanding repertoire of technologies to probe 7TMD protein activities provides the basis upon which to confront functional studies of the uncharacterized receptors in *A. fumigatus* and to screen for inhibitory molecules. It has recently been suggested that considering seven transmembrane receptors as disordered proteins able to allosterically respond to a number of binding partners, is useful in understanding the plasticity of function exhibited by such proteins (Kenakin and Miller, 2010). Conformational changes which occur in response to extracellular ligands and/or stimuli expose cytosolic signaling domains and present three distinct arenas open to perturbation: extracellular sensing/ligand binding, cytosolic surfaces, and intramembrane domains. In order to prioritise the most promising candidates for drug development, a crucial experiment will be to assess the requirement of such receptors, via regulatable promoters, for sustained viability of established fungal mass in murine models of infection (Gossen and Bujard, 1992).

### Conflict of interest statement

The authors declare that the research was conducted in the absence of any commercial or financial relationships that could be construed as a potential conflict of interest.

## Appendix

**Table A1:** **Identity and annotations of *A. fumigatus* proteins having seven predicted transmembrane domains**.

**Annotation**	**ORF**	**Accession number**
**CHROMOSOME 1**		
Polyketide synthase	AFUA_1G01010	XP_749851.1
Conserved hypothetical protein	AFUA_1G01190	XP_749869.1
High affinity zinc ion transporter/membrane zinc transporter	AFUA_1G01550	XP_749905.1
Conserved hypothetical protein	AFUA_1G01620	XP_749912.1
MFS alpha-glucoside transporter	AFUA_1G03280	XP_750078.1
Peroxisomal ABC transporter (PXA1)	AFUA_1G04780	XP_750227.1
Vacuolar membrane PQ loop repeat protein	AFUA_1G06840	XP_750433.1
Phosphatidate cytidylytransferase	AFUA_1G07010	XP_750449.1
Export control protein CHS7-Like	AFUA_1G07110	XP_750458.1
Rhomboid family protein	AFUA_1G09150	XP_752282.1
Conserved hypothetical protein	AFUA_1G10160	XP_752382.1
COP11-coated vesicle protein SurF4/Erv29	AFUA_1G11770	XP_752543.1
PQ loop repeat protein	AFUA_1G11900	XP_752556.1
Integral membrane protein Pth11-like	AFUA_1G14080	XP_752778.1
DUF409 domain protein	AFUA_1G14140	XP_752784.1
DUF803 domain membrane protein	AFUA_1G15880	XP_752954.1
Potassium transporter	AFUA_1G16340	XP_753000.1
Fatty acid elongase (Gig30)	AFUA_1G16710	XP_753038.1
Conserved hypothetical protein	AFUA_1G16720	XP_753039.1
**CHROMOSOME 2**		
RTA1 domain protein	AFUA_2G00420	XP_749178.1
DUF1275 domain protein	AFUA_2G00530	XP_749189.1
Alpha-amylase	AFUA_2G00710	XP_749208.1
Cellobiose dehydrogenase	AFUA_2G01180	XP_749254.1
Bax inhibitor family protein	AFUA_2G03220	XP_749457.1
Extracellular threonine rice protein	AFUA_2G03540	XP_749488.1
ZIP metal ion transporter	AFUA_2G08740	XP_755208.1
Nickel transport protein	AFUA_2G08830	XP_755217.1
Serine/threonine protein kinase	AFUA_2G09570	XP_755291.1
ZIP metal ion transporter	AFUA_2G12050	XP_755537.1
Midasin	AFUA_2G12150	XP_755547.1
Integral membrane protein	AFUA_2G12640	XP_755596.1
HEAT repeat protein	AFUA_2G14180	XP_755751.2
Conserved hypothetical protein	AFUA_2G15100	XP_755844.2
Integral membrane protein	AFUA_2G15440	XP_755879.2
DUF92 domain protein	AFUA_2G15640	XP_755899.1
Rhomboid family membrane protein	AFUA_2G16490	XP_755986.1
Integral membrane protein	AFUA_2G16985	XP_001481687.1
Sulfatase domain protein	AFUA_2G17610	XP_756096.1
Integral membrane protein	AFUA_2G17760	XP_756111.1
RTA1 domain protein	AFUA_2G17810	XP_756116.1
RTA1 domain protein	AFUA_2G17890	XP_756125.1
**CHROMOSOME 3**		
RTA1 domain protein	AFUA_3G00480	XP_748368.1
DUF1275 domain protein	AFUA_3G00670	XP_748388.1
cAMP receptor (Car4)	AFUA_3G00780	XP_748399.1
Hypothetical protein	AFUA_3G00850	XP_748406.2
RTA1 domain protein	AFUA_3G00920	XP_748413.1
Integral membrane protein Pth11-like	AFUA_3G01200	XP_748441.2
RTA1 domain protein	AFUA_3G01630	XP_748484.1
G protein coupled receptor family protein	AFUA_3G01750	XP_748496.1
Conserved hypothetical protein	AFUA_3G02450	XP_748568.1
RTA1 domain protein	AFUA_3G03310	XP_748650.1
PKS-like enzyme	AFUA_3G03540	XP_748674.1
UPF0016 domain protein	AFUA_3G07080	XP_754898.1
Phosphatidylinositol: UDP-GlcNAc transferase PIG-C	AFUA_3G07170	XP_754889.1
Conserved hypothetical protein	AFUA_3G07420	XP_754867.2
DUF1275 domain protein	AFUA_3G07550	XP_754856.1
Sucrose transporter	AFUA_3G08480	XP_754766.1
Conserved hypothetical protein	AFUA_3G09650	XP_754654.1
PQ loop repeat protein	AFUA_3G10470	XP_754572.1
RTA1 domain protein	AFUA_3G10770	XP_754542.1
RTA1 domain protein	AFUA_3G12830	XP_754338.2
Nonribosomal peptide synthase	AFUA_3G13730	XP_754251.1
Mating-type alpha-pheromone receptor PreB	AFUA_3G14330	XP_754193.1
Conserved hypothetical protein	AFUA_3G14870	XP_754138.1
Integral membrane protein	AFUA_3G15100	XP_754114.2
**CHROMOSOME 4**		
Polyketide synthase	AFUA_4G00210	XP_746435.1
Hypothetical protein	AFUA_4G00580	XP_746398.1
Hypothetical protein	AFUA_4G01242	XP_746333.2
Conserved hypothetical protein	AFUA_4G01350	XP_746323.2
Patatin-like serine hydrolase	AFUA_4G03000	XP_746486.1
Aquaporin	AFUA_4G03390	XP_746526.2
Integral membrane protein	AFUA_4G03540	XP_746541.1
C4-dicarboxylate transporter/malic acid transport protein, putative	AFUA_4G04540	XP_746640.1
Para-hydroxybenzoate-polyprenyltransferase Coq2	AFUA_4G05970	XP_752227.1
Longevity-assurance protein (LAC1)	AFUA_4G06290	XP_752195.1
RNA polymerase II mediator complex subunit Nut1	AFUA_4G06600	XP_752166.2
CaaX prenyl protease Ste24	AFUA_4G07590	XP_752066.2
Conserved hypothetical protein	AFUA_4G07680	XP_752057.1
26S proteasome regulatory subunit Rpn2	AFUA_4G08480	XP_751978.1
Conserved hypothetical protein	AFUA_4G10080	XP_751818.2
Endosomal peripheral membrane protein (Mon2)	AFUA_4G12070	XP_751624.1
Potassium uptake transporter	AFUA_4G13540	XP_751477.1
Conserved hypothetical protein	AFUA_4G14210	XP_751411.1
Low affinity iron transporter	AFUA_4G14640	XP_751369.1
**CHROMOSOME 5**		
Integral membrane protein	AFUA_5G00100	XP_748330.2
RTA1 domain protein	AFUA_5G01230	XP_748219.1
RTA1 domain protein	AFUA_5G01310	XP_748211.1
Phosphate permease	AFUA_5G01320	XP_748210.1
Histone acetylase complex subunit Paf400	AFUA_5G02570	XP_748085.1
Integral membrane protein	AFUA_5G02860	XP_748057.2
PQ loop repeat protein	AFUA_5G04100	XP_747934.1
cAMP receptor-like protein	AFUA_5G04135	XP_001481495.1
Conserved hypothetical protein	AFUA_5G06570	XP_753976.1
Integral membrane protein	AFUA_5G06670	XP_753966.1
DUF300 domain protein	AFUA_5G07250	XP_753909.1
a-pheromone receptor PreA	AFUA_5G07880	XP_753848.1
PQ loop repeat protein	AFUA_5G08410	XP_753796.1
Spermine/spermidine synthase family protein	AFUA_5G08500	XP_753787.1
Beige/BEACH domain protein	AFUA_5G09220	XP_753717.1
Bax Inhibitor family protein	AFUA_5G09310	XP_753708.2
RTA1 domain protein	AFUA_5G09900	XP_753650.1
MFS multidrug transporter	AFUA_5G10140	XP_753627.1
MHYT domain signaling protein	AFUA_5G11310	XP_753518.2
26S proteasome regulatory subunit Mts4	AFUA_5G11720	XP_753478.1
Guanine nucleotide exchange factor (Gea2)	AFUA_5G11900	XP_753461.1
Integral membrane protein (Ptm1)	AFUA_5G12390	XP_753413.1
Integral membrane protein TmpA	AFUA_5G12520	XP_753400.1
DUF1275 domain protein	AFUA_5G13060	XP_753348.1
pH signal transduction protein PalH	AFUA_5G13270	XP_753327.1
Integral membrane protein	AFUA_5G13725	XP_753282.2
Integral membrane protein	AFUA_5G14600	XP_753197.1
**CHROMOSOME 6**		
Integral membrane protein	AFUA_6G00320	XP_731523.1
Cation diffusion facilitator	AFUA_6G00440	XP_731511.1
Hypothetical protein	AFUA_6G00460	XP_731509.1
Integral membrane protein	AFUA_6G00640	XP_731492.1
Signal peptide peptidase	AFUA_6G02150	XP_747862.1
Hypothetical protein	AFUA_6G03180	XP_747759.1
Conserved hypothetical protein	AFUA_6G03380	XP_747738.2
Nonribosomal peptide synthase	AFUA_6G03480	XP_747729.1
Integral membrane protein (Pth11)	AFUA_6G03600	XP_747717.1
GTPase activating protein (Tsc2)	AFUA_6G04000	XP_747677.1
Conserved hypothetical protein	AFUA_6G06950	XP_750588.2
IZH family channel protein (Izh3)	AFUA_6G07160	XP_750609.1
4-hydroxybenzoate polyprenyl transferase	AFUA_6G07240	XP_750617.1
Integral membrane protein	AFUA_6G07820	XP_750673.2
Aquaglyceroporin	AFUA_6G08480	XP_750737.1
RTA1 domain protein	AFUA_6G09550	XP_750844.1
Ceramide synthase membrane component (LAG1)	AFUA_6G10460	XP_750934.1
Cell morphogenesis protein (PAG1)	AFUA_6G11010	XP_750987.1
Integral membrane protein	AFUA_6G11560	XP_751039.1
RTA1 domain protein	AFUA_6G11800	XP_751062.1
GPI transamidase component (GAA1)	AFUA_6G12760	XP_751154.1
ABC iron exporter Atm1	AFUA_6G12870	XP_751165.1
UDP-galactose transporter	AFUA_6G13070	XP_751184.1
Ferric-chelate reductase	AFUA_6G13750	XP_751251.1
Integral membrane protein Pth11-like	AFUA_6G13800	XP_751256.1
Integral membrane protein	AFUA_6G13950	XP_751270.1
RTA1 domain protein	AFUA_6G14140	XP_751288.1
**CHROMOSOME 7**		
Plasma membrane hexose transporter	AFUA_7G00220	XP_746907.1
Conserved hypothetical protein	AFUA_7G00280	XP_746901.1
Squalene-hopene-cyclase	AFUA_7G00300	XP_746899.1
Conserved hypothetical protein	AFUA_7G04800	XP_749030.2
Plasma membrane protein Pth11-like	AFUA_7G06130	XP_748897.2
Conserved hypothetical protein	AFUA_7G06660	XP_748845.2
Metalloreductase	AFUA_7G07120	XP_748799.1
**CHROMOSOME 8**		
Solute transporter	AFUA_8G00660	XP_747139.1
Glycosyl transferase	AFUA_8G00680	XP_747137.1
Conserved hypothetical protein	AFUA_8G01300	XP_747076.1
GABA permease	AFUA_8G01450	XP_747061.1
NRPS-like enzyme	AFUA_8G01640	XP_747042.1
Conserved hypothetical protein	AFUA_8G01840	XP_747022.2
Conserved hypothetical protein	AFUA_8G02390	XP_746967.1
ZIP family zinc transporter	AFUA_8G04010	XP_747208.2
Integral membrane protein	AFUA_8G04560	XP_747263.1
Integral membrane protein	AFUA_8G05510	XP_747353.1
Chitin synthase F	AFUA_8G05630	XP_747364.1
RTA1 domain protein	AFUA_8G05740	XP_747375.1
Cellobiose dehydrogenase	AFUA_8G05805	XP_747382.1
DUF1295 domain protein	AFUA_8G05810	XP_747383.2
Toxin biosynthesis protein (Tri7)	AFUA_8G05970	XP_747399.1
Metalloreductase transmembrane component	AFUA_8G06210	XP_747422.2

**Table A2:** **Identity and annotations of *A. fumigatus* histidine kinase receptors**.

**Annotation**	**ORF**	**Accession number**	**No. of transmembrane domains**	**Putative conserved domains**	**Putative group no.**
**CHROMOSOME 2**					
Sensor histidine kinase/response regulator TcsB/Sln1	AFUA_2G00660	XP_001481640.1	4	HiskA (Phospho-acceptor) domain Histidine kinase-, DNA gyrase B- and HSP90-like ATPase Histidine kinase-like ATPases cheY-homologous receiver domain	6
Two-component osmosensing histidine kinase (Bos1)-TcsC	AFUA_2G03560	XP_749489.1	1	Multiple HAMP domains His kinase A (Phospho-acceptor) domain Histidine kinase-like ATPases cheY-homologous receiver domain	3
**CHROMOSOME 3**					
Sensor histidine kinase/response regulator	AFUA_3G07130	XP_754893.1	3	HiskA (Phospho-acceptor) domain Histidine kinase-like ATPases cheY-homologous receiver domain	7
Sensor histidine kinase/response regulator	AFUA_3G12530	XP_754368.1	2	PAS domain HiskA (Phospho-acceptor) domain Histidine kinase-like ATPases cheY-homologous receiver domain	5
Sensor histidine kinase/response regulator	AFUA_3G12550	XP_754366.1	9	Serine/threonine kinase domain AAA ATPase domain GAF domain HiskA (Phospho-acceptor) domain Histidine kinase-like ATPases cheY-homologous receiver domain	10
**CHROMOSOME 4**					
Sensor histidine kinase/response regulator	AFUA_4G00320	XP_746424.2	2	GAF domain HiskA (Phospho-acceptor) domain Histidine kinase-like ATPases cheY-homologous receiver domain	2
Sensor histidine kinase/response regulator	AFUA_4G00660	XP_746390.1	1	GAF domain HiskA (Phospho-acceptor) domain Histidine kinase-like ATPases cheY-homologous receiver domain	2
Sensor histidine kinase/response regulator	AFUA_4G02900	XP_746476.1	2	GAF domain Phytochrome region HiskA (Phospho-acceptor) domain Histidine kinase-like ATPases cheY-homologous receiver domain	8
Sensor histidine kinase/response regulator	AFUA_4G01020	XP_746355.1	3	GAF domain HiskA (Phospho-acceptor) domain Histidine kinase-like ATPases cheY-homologous receiver domain	2
Sensor histidine kinase/response regulator	AFUA_4G07400	XP_752086.2	0	Histidine kinase-like ATPases cheY-homologous receiver domain	7
**CHROMOSOME 6**					
Sensor histidine kinase/response regulator Fos-1/TcsA	AFUA_6G10240	XP_750913.1	1	PAS domain HiskA (Phospho-acceptor) domain Histidine kinase-like ATPases cheY-homologous receiver domain	5
**CHROMOSOME 8**					
Sensor histidine kinase/response regulator	AFUA_8G06140	XP_747415.2	2	HiskA (Phospho-acceptor) domain Histidine kinase-like ATPases cheY-homologous receiver domain	7

HiskA, Histidine kinase A; HAMP, Histidine kinases, adenyl cyclases, methyl-accepting chemotaxis protein, phosphatase; PAS, Per—period circadian protein, Arnt—Al receptor nuclear translocator protein, Sim—single minded protein; GAF, presence in cGMP-regulated cyclic nucleotides PDEs, certain adenyl cyclases and the bacterial transcription factor FhlA; AAA, ATPases associated with diverse cellular activities.
